# Synthesis and evaluation of the bioactivity of fluorapatite–45S5 bioactive glass nanocomposite

**DOI:** 10.1007/s40204-019-0112-y

**Published:** 2019-04-11

**Authors:** Sahebali Manafi, Fatemeh Mirjalili, Rayhaneh Reshadi

**Affiliations:** 1 Department of Materials Engineering, Shahrood Branch, Islamic Azad University, Shahrood, Iran; 2 Department of Materials Engineering, Maybod Branch, Islamic Azad University, Maybod, Iran; 3 Department of Materials Engineering, Yazd Branch, Islamic Azad University, Yazd, Iran

**Keywords:** Bioactive glass 45S5, Fluorapatite, Bioactivity, X-ray powder diffraction, Sol–gel

## Abstract

This research study concerns the evaluations of nano-biocomposite ceramics’ characteristics and biocompatibility. A nanocomposite with 45S5 bioactive glass base has been synthesized by sol–gel method. The synthesized nanocomposites were characterized with the help of different techniques, using field-emission scanning electron microscope, X-ray powder diffraction, energy-dispersive X-ray spectroscopy to evaluate the crystal structure, microstructure, and the morphology of the nanocomposite. The results indicated that the synthesis of 45S5 bioactive glass–fluorapatite nanocomposites produced an average particle size of about 20–30 nm and percentages of crystallinity of about 70–90%. fluorapatite–45S5 bioactive glass nanocomposites were characterized in terms of their degradation by determining the weight change percentages, pH changes, the ion release and in terms of bioactivity by checking the apatite layer formation using a solution of simulated body fluid (SBF). The results showed non-cytotoxicity and the formation of a thick apatite layer on the synthesized nanocomposites within 28 days after soaking in SBF. This is an indication of desirable bioactivity in the synthesized particles.

## Introduction

Nowadays, biocompatible synthetic materials are used to exchange with injured tissues. However, due to slight similarities between the chemical, biological, or physical properties of these materials and the host tissue, there are often failures which require retreatments (Soundrapandian 2010).

The limitations of conventional processes such as autograft, allograft, and xenograft for this determination include low availability, lack of adequate mechanical strength and configurability, transmitting viruses, and causing illnesses in the recipient. Considering the serious arguments regarding the use of natural bone grafts, the development of synthetic materials made of metals, ceramics, polymers, and composites for bone substitution is highly crucial (Hench 2006). The fundamental feature of metal alloys is their appropriate mechanical property. However, there are always concerns about their bioactivity and aptitude to junction with living tissue without external forces and resistance to their corrosion in physiological environment (Mancuso 2017). Contrary to metal alloys, there are known biocompatible ceramics and glasses with outstanding biological properties that create a strong chemical bond with tissues in a short time (Kheradmandfard 2010) and show suitable biological answer in the junction of bone and tissue and thus have extensive applications in medicine (Kokubo 2006; Shankhwar and Srinivasan 2015). However, due to their unsuitable physical properties, their demand is faced with problems in sections under mechanical load (Jmal and Bouaziz 2017).

The maximum content of the mineral composition of natural bone is comprised of hydroxyapatite [Ca_10_(PO_4_)_6_(OH)_2_] nanocrystal structure (Dorozhkin 2016). Several studies have reported that calcium phosphates and their complexes such as hydroxyapatite can present a desired environment for bone tissue regeneration due to their likeness to the chemical composition of natural bone, tooth, and enamel (Manafi and Joughehdoust 2009; Stabile 2016; Chen 2017; Khoshakhlagh 2017; Resmim et al. 2018). It was found that the addition of fluoride ions into hydroxyapatite structure increases its resistance to biodegradability significantly (Joughehdoust et al. 2010). In addition, by better absorption of protein, it expresses a stronger cellular connection and reinforces the activity of phosphates to create stronger osteoconductivity (Barandehfard 2016; Santos 2016). When OH^−^ groups in HA are completely exchanged with F^−^ groups, fluorapatite [Ca_10_(PO_4_)_6_F_2_] is formed (Shen 2016), which has more chemical and structural constancy compared with hydroxyapatite (Joughehdoust et al. 2010; Sumathi 2015; Shen 2016). This ion replacement makes positive impact on proliferation, morphology, and differentiation of osteoblast-like cells and improves bioactivity (Roche 2014). There is 1 wt% fluorine in the cortical bone. The presence of this level of fluorine in the bone stops loss of bone density, which is the aim of osteoporosis (Zhao 2014). Fluorapatite also forms the outer layer of tooth (Stanic 2014). The mineral phase of tooth under the enamel covers about 0.04–0.07 wt% fluorine (Tredwin 2013). 45S5 bioglass is a distinguished biomaterial due to its unique set of properties such as unmatchable biocompatibility, outstanding bioactive performances, and antibacterial characteristics (Baino et al. 2018). The first reported bioactive glass (BG) was created by Professor Larry Hench in 1969 with a composition of 45 wt% SiO_2_, 24.5 wt% Na_2_O, 24.5 wt% CaO, and 6 wt% P_2_O_5_ (Cooper 2006). This discovery was also the beginning of the second generation of biomaterials that have the ability to bond with host tissues (Willigeroth 2002).

The most amazing feature about the BG finding was its ability to improve very strong interfacial bonds with the surrounding tissues (Joughehdoust and Manafi 2012). Moreover, BG displays the highest in vivo bioactivity index among all bioceramics (Rodriguez-Lorenzo and Gross 2003). In spite of its surprising biocompatibility and the bone-bonding capability, BG has limited requests as a scaffold material due to its poor mechanical properties (Loher 2005). The mixture of bioactive glass particles with fluorapatite affords it special properties such as augmentation of bioactivity and mechanical properties. The purpose of this study is to produce fluorapatite–bioactive glass and comparing its properties with hydroxyapatite/bioactive glass. The present study aims to synthesize and characterize fluorapatite–45S5 bioactive glass nanocomposite by sol–gel method for bone tissue and evaluating their bioactivity using in vitro method. The novelties of this research are the effect of different percentages of fluorapatite for preparing the fluorapatite–bioactive glass 45S5 nanocomposite and investigation of the morphological and microstructural properties. In addition, fluorapatite–45S5 bioactive glass nanocomposites were characterized in terms of degradation by determining the weight change percentages, pH changes, ion release and in terms of the bioactivity by checking the apatite layer formation using a solution of simulated body fluid (SBF). The target of this project is to develop and evaluate a nanosized glass–ceramic composite with the properties close to bone and good biocompatibility for application in dentistry and the orthopedics as a bone constructor.

## Materials and methods

### Preparation of bioactive glasses of 45S5–fluorapatite nanocomposite

For synthesis of bioactive glasses of 45S5–fluorapatite nanocomposites with 10, 15, and 20% wt% of fluorapatite, two sols of bioactive glass and fluorapatite were prepared. The sol of bioactive glasses 45S5 was prepared by hydrolysis of 14.54 mL tetraethoxy silane (TEOS) in 350 mL of distilled water and 350 mL ethanol at room temperature and the pH was adjusted at 2 by nitric acid with continuous stirring for 1 h. In the next step, 8.97 g hydrated calcium nitrate was added into the above solution and continued stirring until its dissolution. After that, 1.23 g NaNO_3_ was added into the above mixture (the previous mixture was named solution A), and then, 1.23 g ammonium dehydrogenase phosphate was added into distilled water (this mixture was named solution B). Furthermore, solution (B) was gradually added to solution (A) with overnight continuous stirring.

For preparation of fluorapatite sol, at first, 11.82 g hydrated calcium nitrate [Ca(NO_3_)•4H_2_O] was dissolved in 30 mL absolute ethanol/water, and also, another solution was made by dissolving 2.13 diammonium hydrogen phosphate [(NH_4_)_2_HPO_4_] in 30 mL ethanol/distilled water. Both solutions were stirred for 1 h to obtain transparency.

In the second step, the aqueous solution was added dropwise at a rate of 5 mL min^−1^ to the alcoholic solution with vigorous stirring.

The pH of the solution was regulated to 10 by dropwise addition of NH_4_OH. To synthesize fluorapatite (FA), the solution of hydrated calcium nitrate and diammonium hydrogen phosphate, and the solution of 0.56 g ammonium fluoride (NH_4_F) with 30 mL ethanol/water was added and stirred.

Then, the two sols were combined and stirred for 2 h using a magnetic stirrer. The obtained sol was maintained at ambient temperature with a different weight ratio of fluorapatite for 14 days until it became a uniform and transparent gel. The obtained gels were centrifuged and washed by ethanol four times and dried at 80 °C for 5 h, and subsequently grinded with mortar and pestle. Finally, the resulting fine nanocomposite powder was sintered at 600 °C for 1 h.

### Characterization of bioactive glasses of 45S5–fluorapatite nanocomposite

Phase identification was performed by X-ray diffraction (XRD) PW1800, of Philips Company (USA), using nickel filtered CuK_α_ radiation in the range of 2*θ* = 10°–60° with a scanning speed of 5° per minute. The crystallite size was determined by the Scherrer method. The equation was calculated as follows:

1 $$t = {\raise0.7ex\hbox{${0.89\lambda }$} \!\mathord{\left/ {\vphantom {{0.89\lambda } {\beta \cos \theta }}}\right.\kern-0pt} \!\lower0.7ex\hbox{${\beta \cos \theta }$}},$$

where *t* is the crystallite size (in nm), *λ* = wavelength of X-ray diffraction (in nm), beta = full width at half-maximum, and theta = Bragg diffraction angle (Lindfors 2010; Kolk 2012).

The crystallinity degree of the hydroxyapatite phase was calculated using X-ray diffraction patterns according to the following equation:

2 $$X_{\text{c}} = 1 - (V_{112/300} - I_{300} ),$$

where *X*_c_ = degree of crystallinity of powder and $V_{112/300}$ = intensity of the cavity between diffracted peaks (112) and I300 (Rahaman 2011).

A Fourier transform infrared spectrometer (FTIR, Perkin Elmer Spectrum 100, USA) was used with the universal attenuated total reflection (UATR) method. The microstructures of the powders were identified by transmission electron microscopy (TEM, Philips-Zeiss-Germany) and scanning electron microscopy (SEM PHENOM, Hitachi, Japan) and field-emission scanning electron microscopy (FESEM, Hitachi S-2460 N, Japan).

### External evaluation of synthesized material in SBF solution

To prepare 1 L of a simulated body solution (SBF), 700 mL deionized water was stored in the hot water bath. Sodium chloride salts (NaCl), sodium bicarbonate (NaHCO_3_), potassium chloride (KCl), potassium hydrogen phosphate tri-acetic acid (K_2_HPO_4_•3H_2_O), hydrated magnesium chloride (MgCl_2_•6H_2_O), 1 molar hydrochloric acid, calcium chloride (CaCl_2_), and sodium sulfate (Na_2_SO_4_) would be dissolved in deionized water and reached the desired temperature. Upon completion of the combination of these salts, again, using deionized water that was also heated with salt solution and the volume of this solution increased to 900 mL. The solution temperature at the end should be about 36 °C and its pH was less than 2. After controlling the temperature and the pH, in the next step, the pH was increased to 7.45. When the pH reached 4.45, 1 M hydrochloric acid was added until it reached 7.42. The volume of this solution was brought to the end by deionized water to 1000 mL (Gosain and Plastic Surgery Educational Foundation DATA Committee 2004; Giannoudis and Dinopoulos 2005).

### Bioaccumulation and biodegradability test

To evaluate the external properties of the nanocomposites, the synthesized powders were immersed in a simulated fluid in different time intervals. The synthesized nanopowders and the simulant solution were mixed with 1 mg/mL ratio and then placed on the shaker at 37 °C.

The procedure was to ensure that the specimens were stored in a solution for a specified period. During this period, this solution changed over a specified period of time. Next, the sample was analyzed for concentration testing, and the specimen was washed after the exhaustion and heated to 50 °C for complete drying and subjected to additional measurements. To study the biodegradability and bioactivity of the produced samples, after the immersion of the samples, various parameters such as pH change and weight change were calculated according to the following equation:

3 $${\text{weight loss}} \left( \% \right) = {\raise0.7ex\hbox{${\left( {M_{\text{bi}} - M_{\text{af}} } \right)}$} \!\mathord{\left/ {\vphantom {{\left( {M_{\text{bi}} - M_{\text{af}} } \right)} {M_{\text{af}} }}}\right.\kern-0pt} \!\lower0.7ex\hbox{${M_{\text{af}} }$}} \times 100,$$

where *M*_bi_ is the initial weight and *M*_af_ is the weight after immersion. In addition, the concentration of ions released from the synthesized materials and also the surface of these specimens was investigated for the possible formation of apatite layer. The following techniques were used for this purpose.

To study the concentration of released fluoride ion in fluorinated ion-containing synthesized samples, a fluoride ion electrode (Fluoride ISE Metrohm) in stimulant solution was used. Therefore, to evaluate the process of formation of the apatite layer on immersed samples, analyses with electron microscopy and X-ray diffraction energy were performed.

## Results and discussion

### X-ray diffraction analysis

**Fig. 1:**
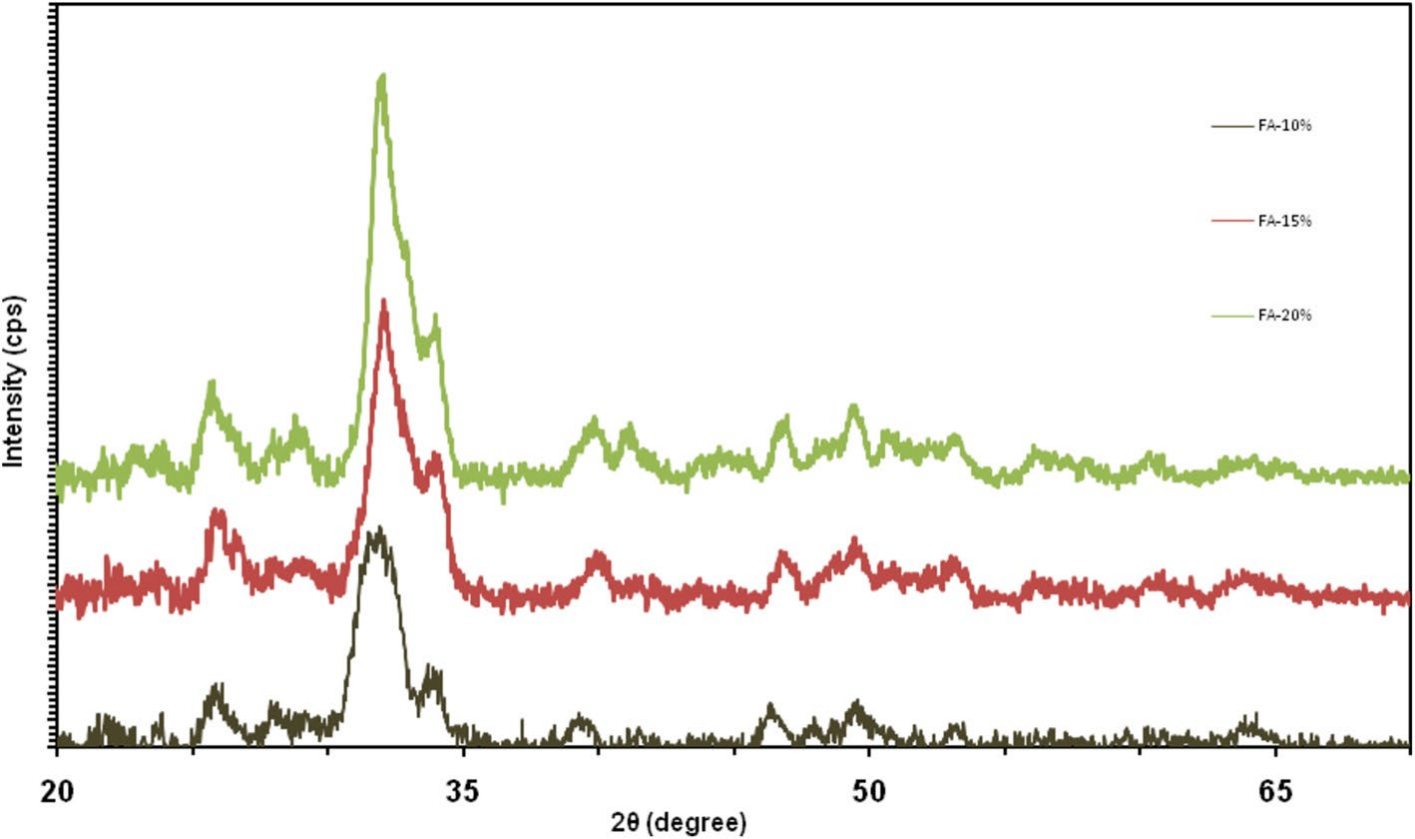
X-ray diffraction pattern of bioactive glass–fluorapatite nanocomposites containing 10% to 15–20 wt% of fluorapatite

Figure 1 shows the X-ray diffraction pattern of the bioactive glass–fluorapatite composite samples. The synthetic nanosized particles were distinctly distinguished from the synthesized composite dispersion pattern. The presence of glass in the material structure has led to the expansion of diffraction patterns of the materials. The XRD patterns show that the intensity of fluorapatite diffraction peaks was increased which confirmed the nanosize with crystalline nature. The particle size and crystallite percentage of the nanocomposites are displayed in Table 1. As it is shown, by increasing the fluorapatite from 10 to 20% the particles were grown and the crystallinity was developed.

**Table 1:** Comparison of particle size and crystallite percentage of nanocomposites

Sample (% FA)	Particle size (nm)	Crystallinity (%)
10	21	73
15	25	79
20	29	88

### Morphological investigation of nanocomposites

**Fig. 2:**
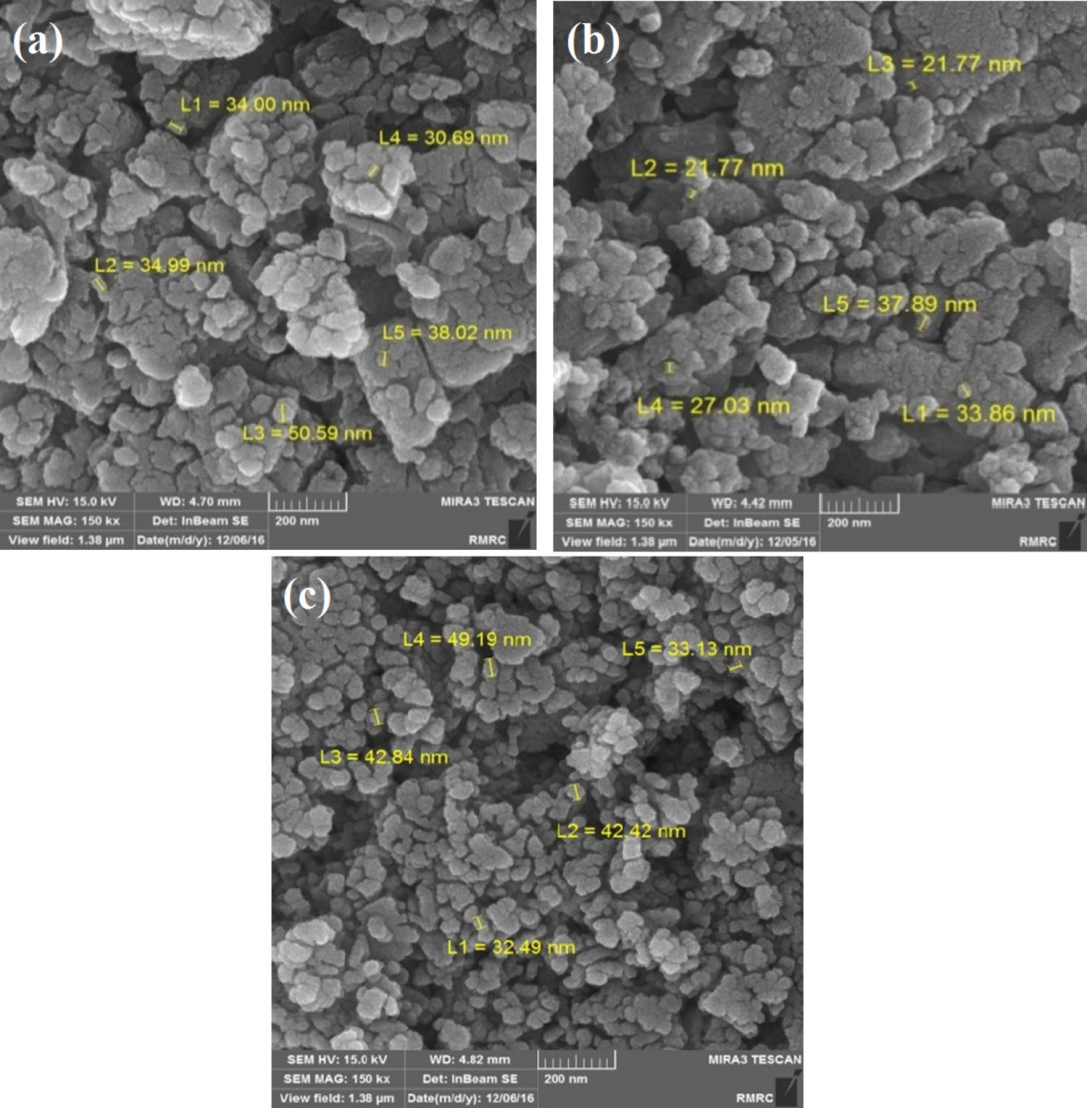
Field-emission scanning images of bioactive glass–fluorapatite nanocomposites **a** 10%, **b**15%, **c** 20%

Field-emission scanning electron microscopy (FESEM) images of 45S5 bioactive glass–fluorapatite nanocomposites are exhibited in Fig. 2. The spherical shape of the powders with size range of about 20–50 nm confirms the results of XRD.

### Evaluation of the bioactivity in SBF

#### Degradability and biocompatibility

To study the biocompatibility and biodegradability analyses of the samples, after SBF immersion and ion concentration from the SBF solution, some tests were performed as follows:

#### Weight changes

**Fig. 3:**
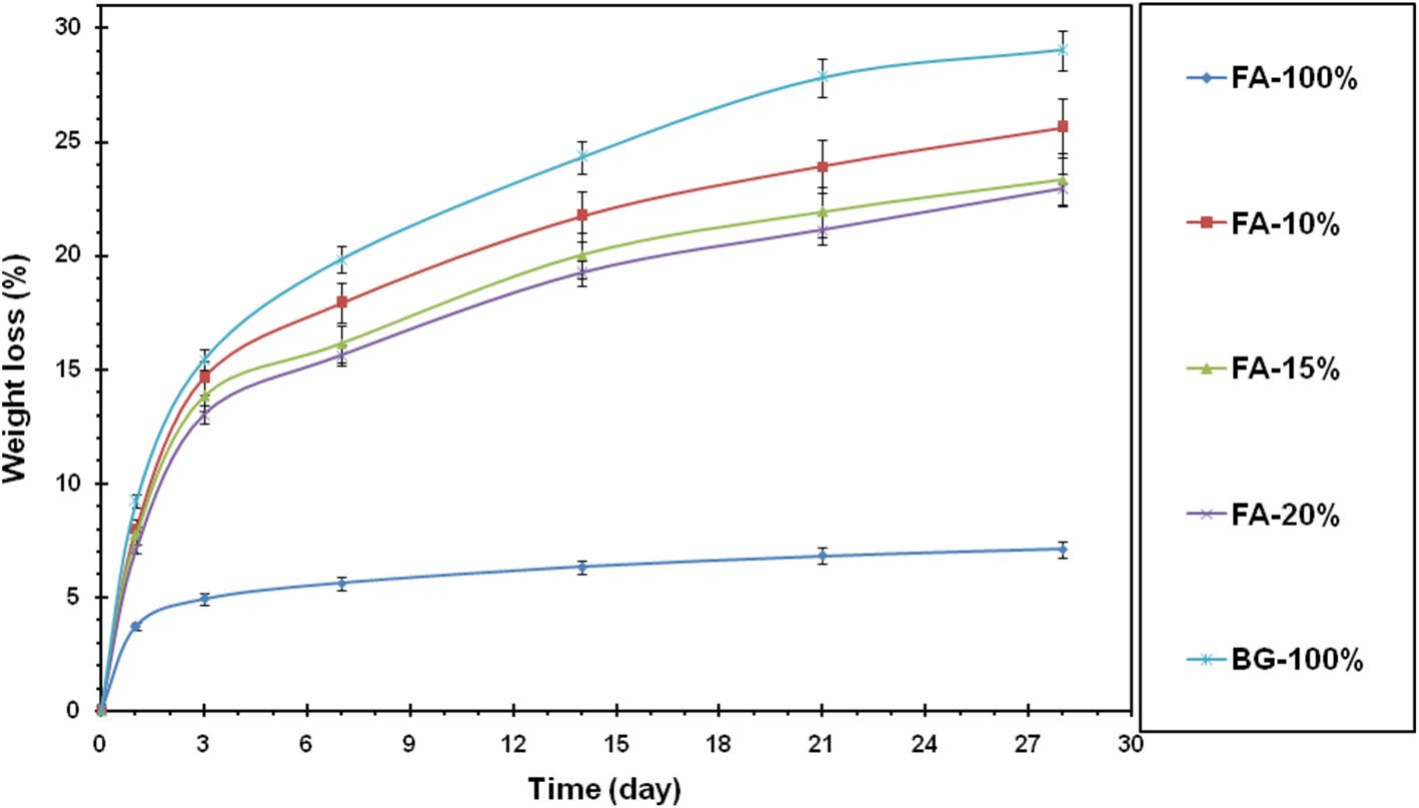
Weight change percentage of nanocomposites in SBF

After immersion of the specimens in SBF solution, the weight change of the samples was calculated. As it was shown in the diagram in Fig. 3, samples have undergone rapid weight loss in the early days and have been relatively stable since the 7th day. In the early days, the samples were washed by immersion in the solution. By immersion in the solution, the apatite layer was formed on surfaces of the samples and ultimately stabilized by repeating this flow of apatite layer until no weight changes. The stability of calcium phosphate phases significantly depended on the temperature. Each calcium phosphate was able to convert to hydroxyapatite in the presence of sufficient water which was due to the presence of calcium phosphate compounds in the synthesized composite sample. When this sample was placed adjacent to the body fluid simulator, this hydroxyapatite layer began to form. The activation started in the solution and the pH was changed severely with the formation of apatite layer. Moreover, due to the presence of fluorine ion in the fluorapatite structure, this material had lower solubility. In addition, it could be expected that fewer fluoride composites have higher bioavailability which could release more Si in the early days.

#### pH Changes

During the initial hours and early days, the ion exchange occurred by immersion in the simulator solution. Positive cations such as calcium and sodium as well as positive hydrogen were replaced in the environment. In addition, phosphate and fluorine ions replaced the hydroxyls, and the ion exchange inside the solution changed significantly. Thus, in the early days and immediately after immersion of the samples in the simulator solution, the release of the elements of the samples was larger and this process created a great change.

**Fig. 4:**
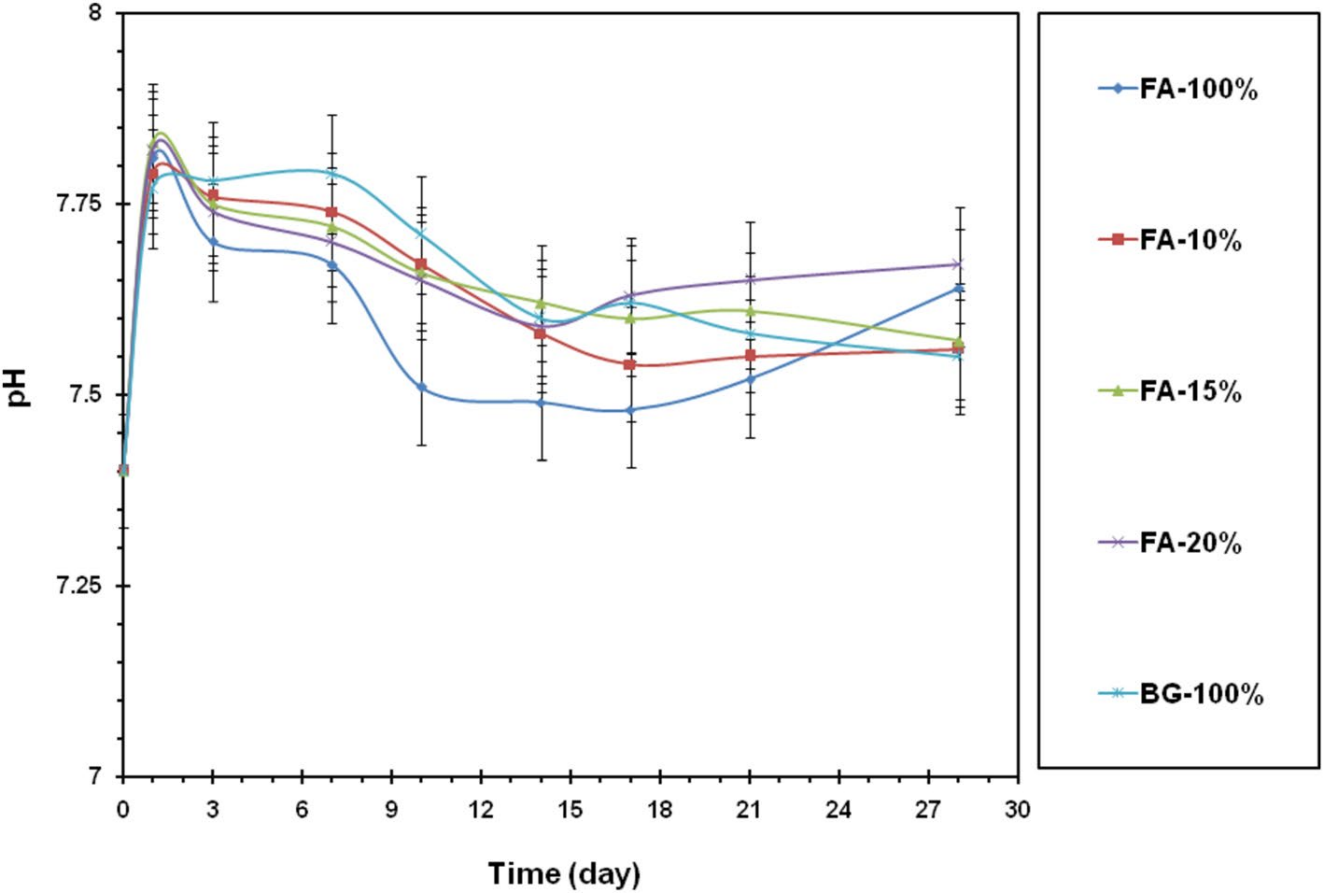
pH changes in SBF solution

By forming a bioactive layer on the samples, this release was stabilized and prevented a sudden change in the pH of the solution. When the apatite diameter was added, the process became more uniform. Therefore, the effect of the presence of fluorine ion on pH changes was clearly visible in the graph in the Fig. 4.

In the early days, the bioactive glass–fluorapatite nanocomposites were effective in increasing the pH compared to the sample containing pure 45S5 bioactive glass. Due to the presence of the ionic fluorine, instead of forming a layer of hydroxyapatite, the fluorapatite layer would be formed on the surface, which showed lower solubility than hydroxyapatite layer. In addition, because it had a calcium phosphate phase in the composition, the increase in pH on the first days had a direct effect on the sample’s solubility which showed that the absorption process would be accelerated when the sample is in the body. It could be expected that on the first days, this sample would be able to stimulate osteogenesis. The sample containing 100% fluorapatite in the last days had again increased the pH, which might resume resumption of the hydroxyapatite layer by delivery of the ion. In general, from the chart, it could be seen that, at the initial days, the sample in a physiological environment almost changed completely; in addition, the same could be concluded from reviewing the pH graph and the weight change chart. Since the curve of the sample contained 15% fluorapatite, the change in weight loss was less and the pH was more stable. Therefore, the formation of the apatite layer in this sample was more than other samples. For a sample containing 10% fluorapatite after day 21, the pH was increased, which could be attributed to the beginning of sample’s disappearance in the SBF.

#### Ion delivery

**Fig. 5:**
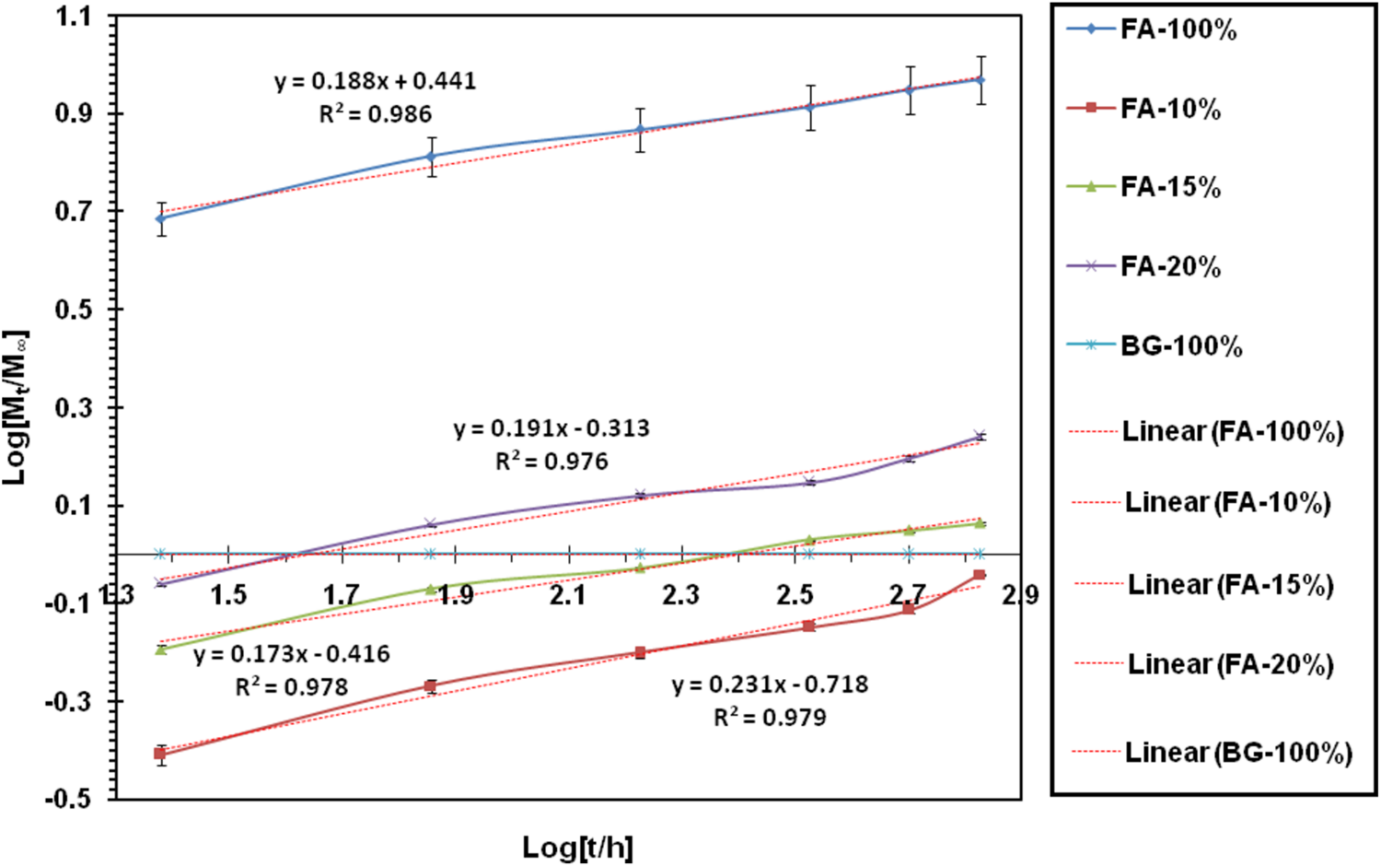
Fluoride ion release from nanocomposites in SBF

**Fig. 6:**
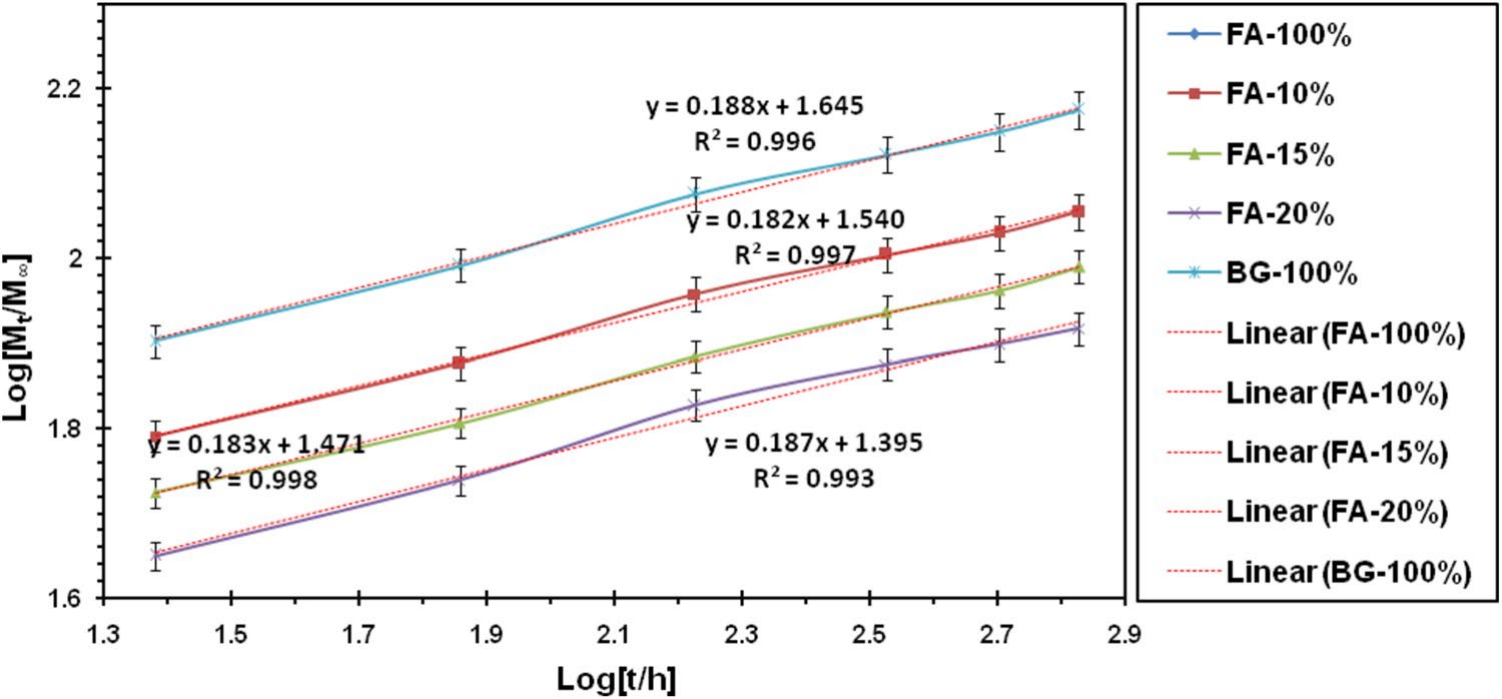
Si release from samples in SBF

The release graph of fluoride and silicon ions is shown in Figs. 5 and 6. Immediately after the placement of the synthesized sample in the physiological environment, the ion exchange would begin with its surroundings and changed the ions’ concentration. As it was clear in its release diagram, two elements were considered as representative of two materials in the composite. Since F and Si elements were not available from the beginning in the SBF solution, the change in the concentration of these two elements was a good criterion for evaluating the biodegradability of the composites.

The produced composites were capable to increase bioactivity in the vicinity of body physiological environment which was due to ion release of Si^4+^ ion. The release rate of the ions with its release and by creating the apatite layer and increasing its diameter in the simulator solution was directly related to the original sample.

The Korsmeyer–Peppas model of the material release mechanism was a combination of the simple diffusion of the Fick and Type II, which considered the relationship between the time and the released ion. Therefore

4 $$\frac{{M_{t} }}{{M_{\infty } }} = kt^{n} ,$$

in which *M*_*t*_/*M*_∞_ was the fraction of the released ion at time *t*, *k* was the speed constant, and *n* was its exponent of release.

Therefore

5 $${\text{Log}}\left( {\frac{{M_{t} }}{{M_{\infty } }}} \right) = {\text{Log}}k + n{\text{Log}}t.$$

According to the above relationship, Log (*M*_*t*_/*M*_∞_) in terms of log *t* was a straight line, whose slope denoted the value of *n*, and formed the origin of the line, it was possible to determine the rate of its release (Muhammad et al. 2018).

The *n* value obtained from this relationship has been used to discuss its release kinetics.

Accordingly, different values of *n* could be attributed to different release mechanisms. Table 2 shows the classification in this area.

**Table 2:** Classification of ion release mechanisms based on the value of its representation of* n* according to Korsmeyer–Peppas model

Fick diffusion	*n *= 0.45
UnFick diffusion	0.45 < *n *< 0.89
Transition (case-II)	*n *= 0.89
Supertransition (case-II)	*n *> 0.45

In Table 2, the values *n* and *k* for the synthesized materials were presented.

According to these values and based on the Korsmeyer–Peppas model, the release mechanism of the ion was on diffusion model and following the Fick rules.

Consequently, the following points could be prepared. According to the results of Table 3, the highest ion release rate of fluoride in the samples was related to a pure fluorapatite and the highest fixed-release rate of the silicon ion in the samples was related to a pure bioactive glass. It could be stated that the constant ion release rate was directly related to the presence of the ion in the initial material. Reducing the release rate of each ion in the composite samples was due to the reduction of the ion presence in the material, and the second component of the composite had no effect on its ion release. Among the synthesized composites, FA-15% nanocomposite was an optimal sample among the composites, it was due to its ion release process and also the biodegradability and biodegradability results.

**Table 3:** Calculated values of *n* (exponent of release) and *k* (release rate constant) for different samples

Release of (Si^4+^)		Release of (F^−^)		Sample (%)
*k*	*n*	*k*	*n*	
0.000	0.000	2.761	0.189	FA-100
24.84	0.188	0.486	0.191	FA-20
29.58	0.183	0.384	0.174	FA-15
34.73	0.183	0.191	0.232	FA-10
44.20	0.188	0.000	0.000	BG-100

### Morphological results after immersion in SBF

**Fig. 7:**
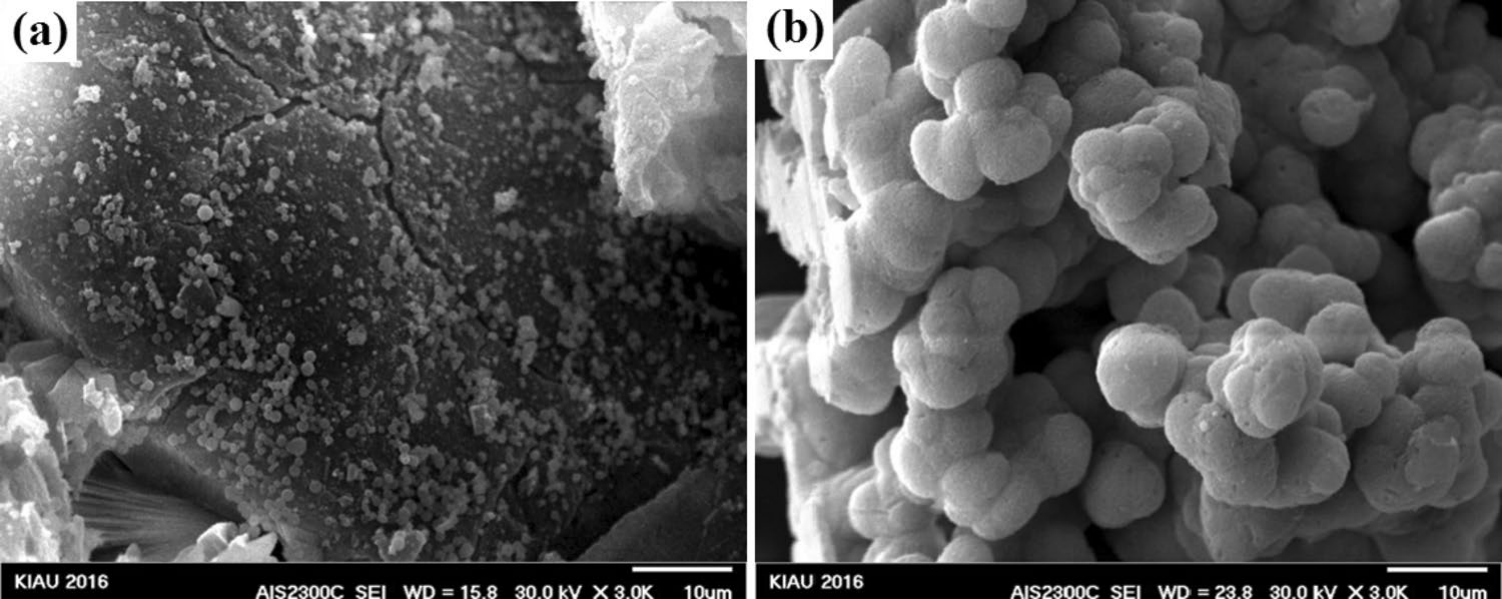
Scanning electron microscopy images of the bioactive glass nanoparticles after 1 (**a**) and 28 (**b**) days immersion in SBF

**Fig. 8:**
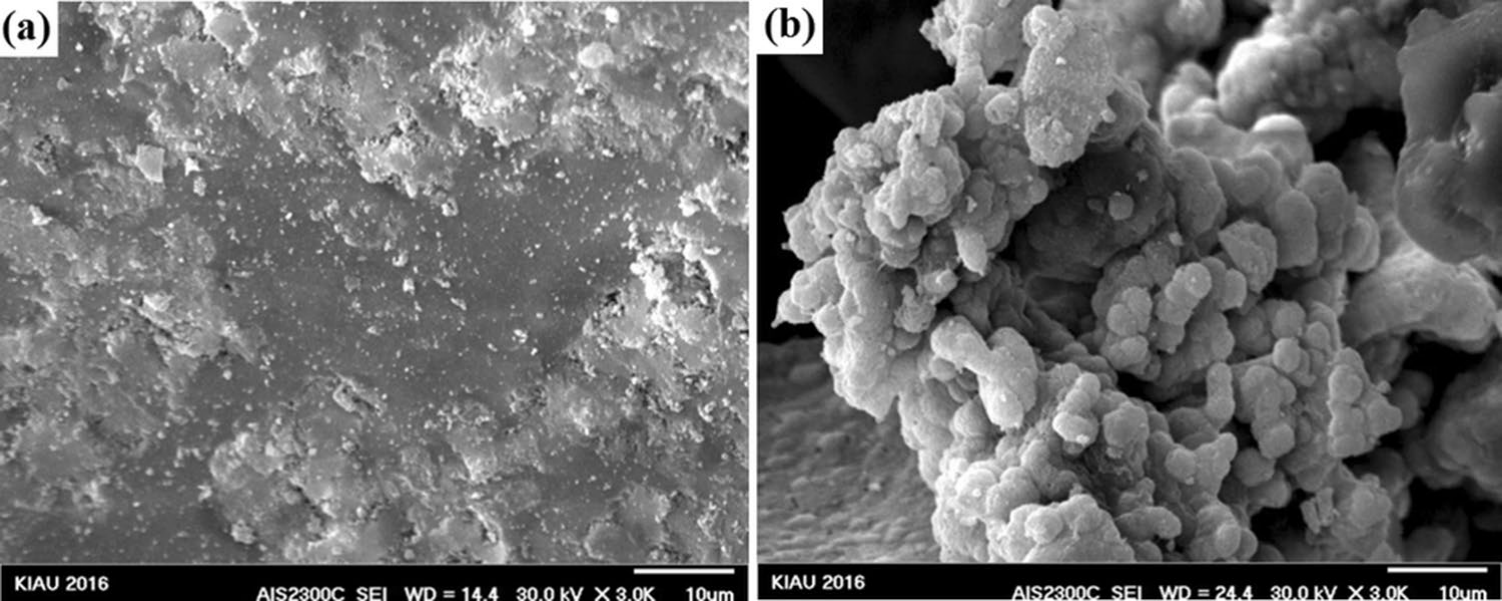
Scanning electron microscopy images of bioactive glass–fluorapatite nanocomposite containing 10% by weight of fluorapatite after 1 (**a**) and 28 (**b**) days immersion in SBF

**Fig. 9:**
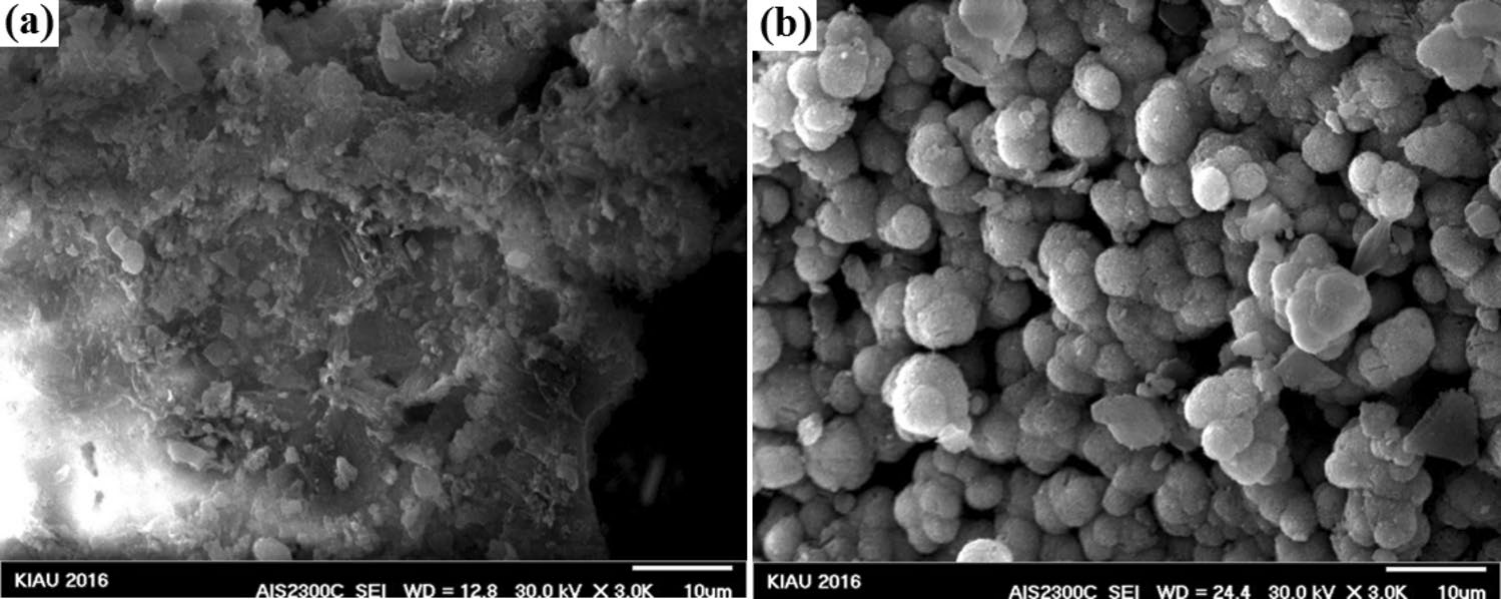
Scanning electron microscopy images of bioactive glass–fluorapatite nanocomposite containing 15% by weight of fluorapatite after 1 (**a**) and 28 (**b**) days immersion in SBF

**Fig. 10:**
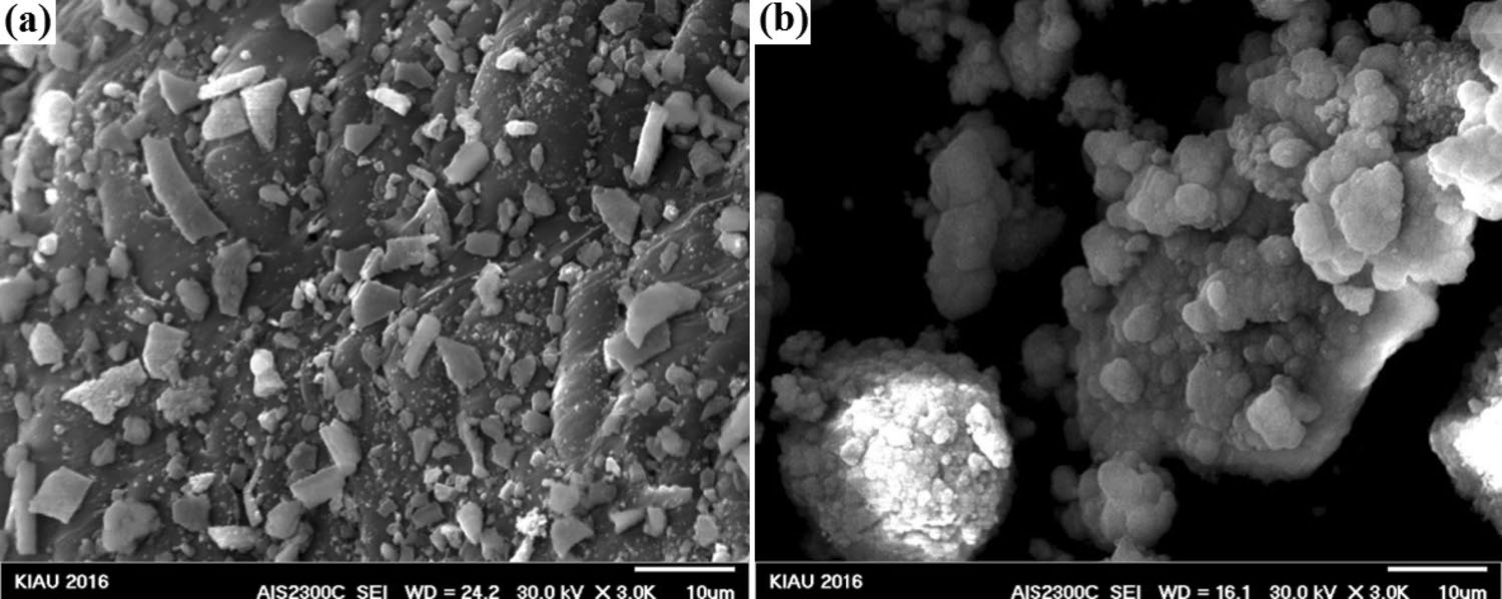
Scanning electron microscopy images of bioactive glass–fluorapatite nanocomposite containing 20% by weight of fluorapatite after 1 (**a**) and 28 (**b**) days immersion in SBF

**Fig. 11:**
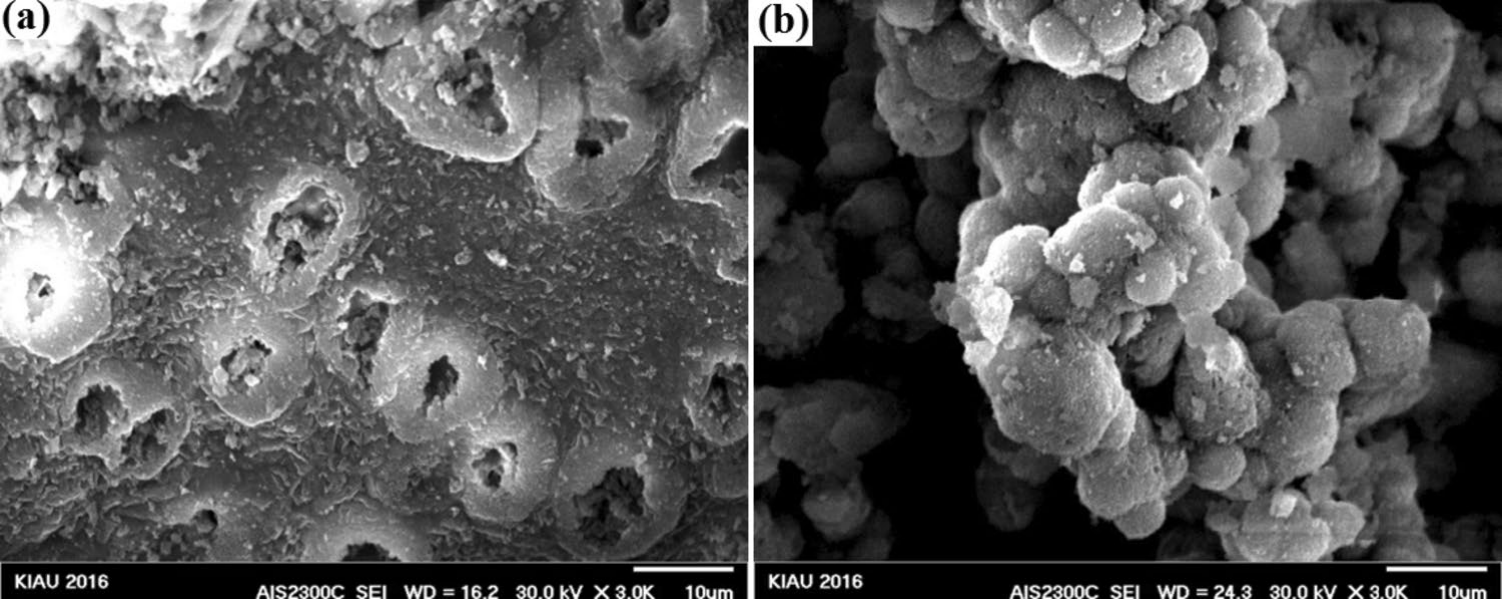
Scanning electron microscopy images of fluorapatite nanoparticles after 1 (**a**) and 28 (**b**) day immersion in SBF

FESEM results of the synthesized nanocomposites after immersion in SBF solution are shown in Figs. 7, 8, 9, 10, and 11. To investigate the bioactivity trend of the samples on the first and last days, these images were compared. The HA particles on the surface of two images were developed. However, on the last day, the polygonal apatite crystals which were more likely to form fluorapatite crystals were appeared and fully covered them.

These concepts indicated that the growth of these crystals as a result of the formation of a hydroxyapatite layer on the surface of the samples confirms the bioactivity of the samples. It is noticeable that the sample containing the highest amount of fluorapatite exhibited less porosity on the surface.

#### SEM–EDX results after immersion in SBF solution

**Fig. 12:**
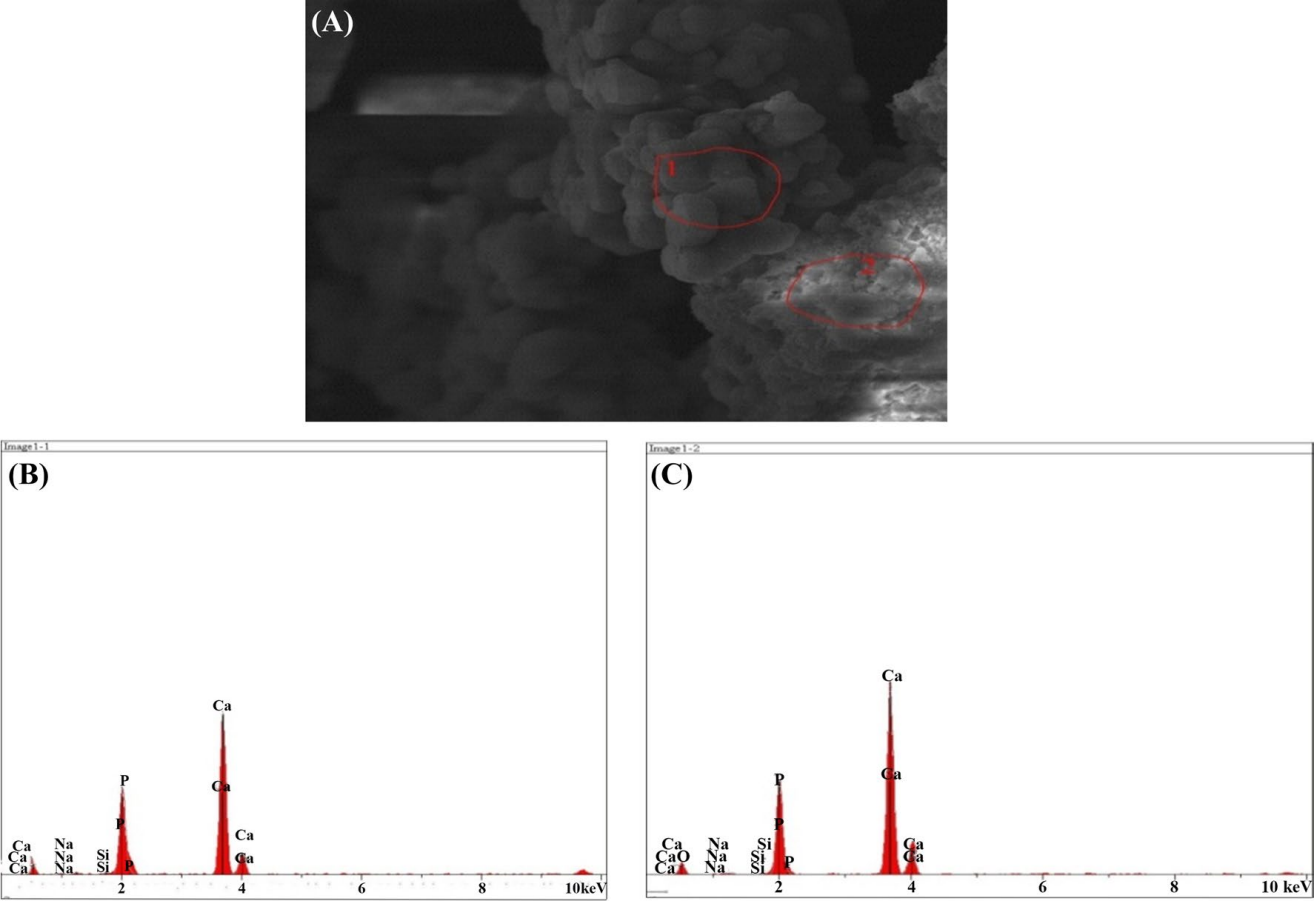
**a** SEM and **b**, **c** EDX images of the bioactive glass

**Fig. 13:**
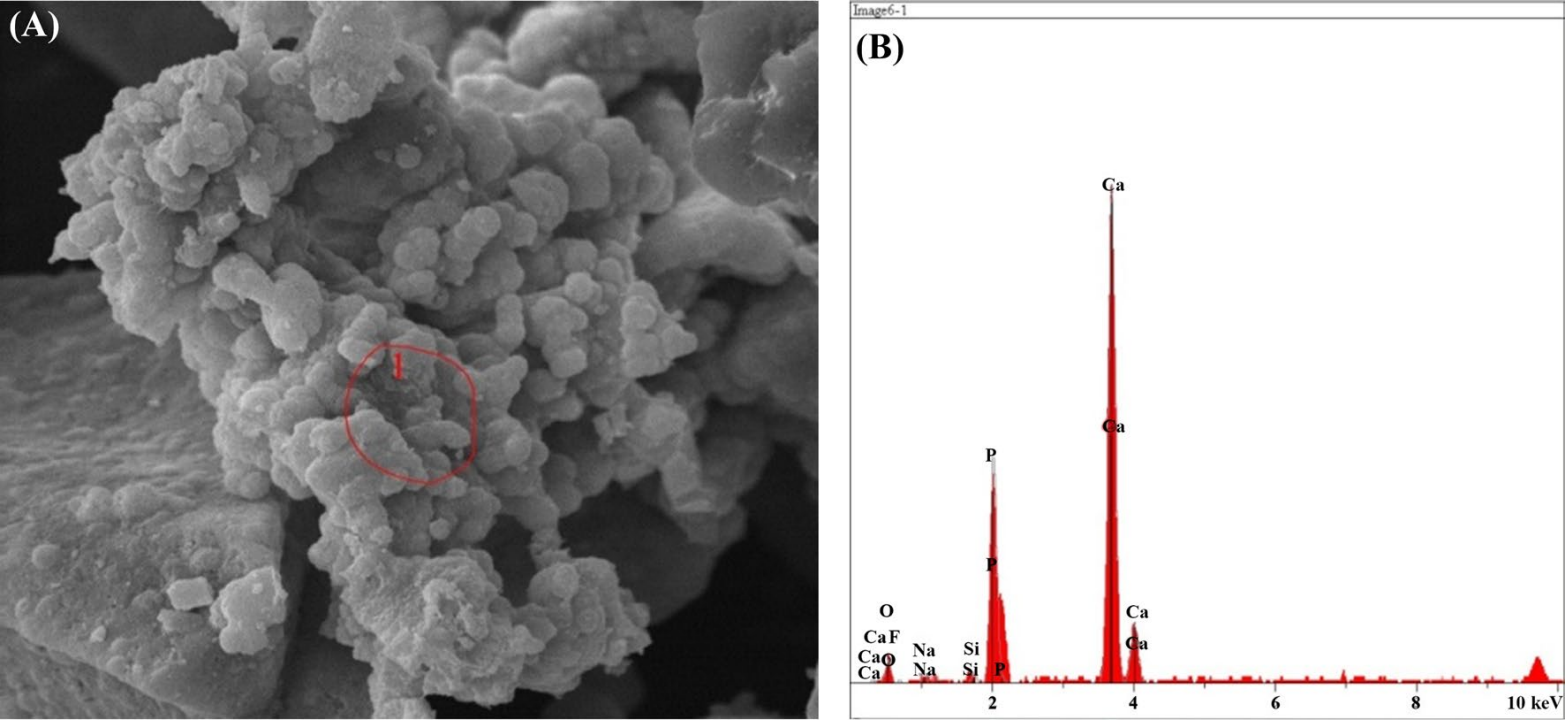
**a** SEM, **b** EDX images of the sample containing 10% fluorapatite

**Fig. 14:**
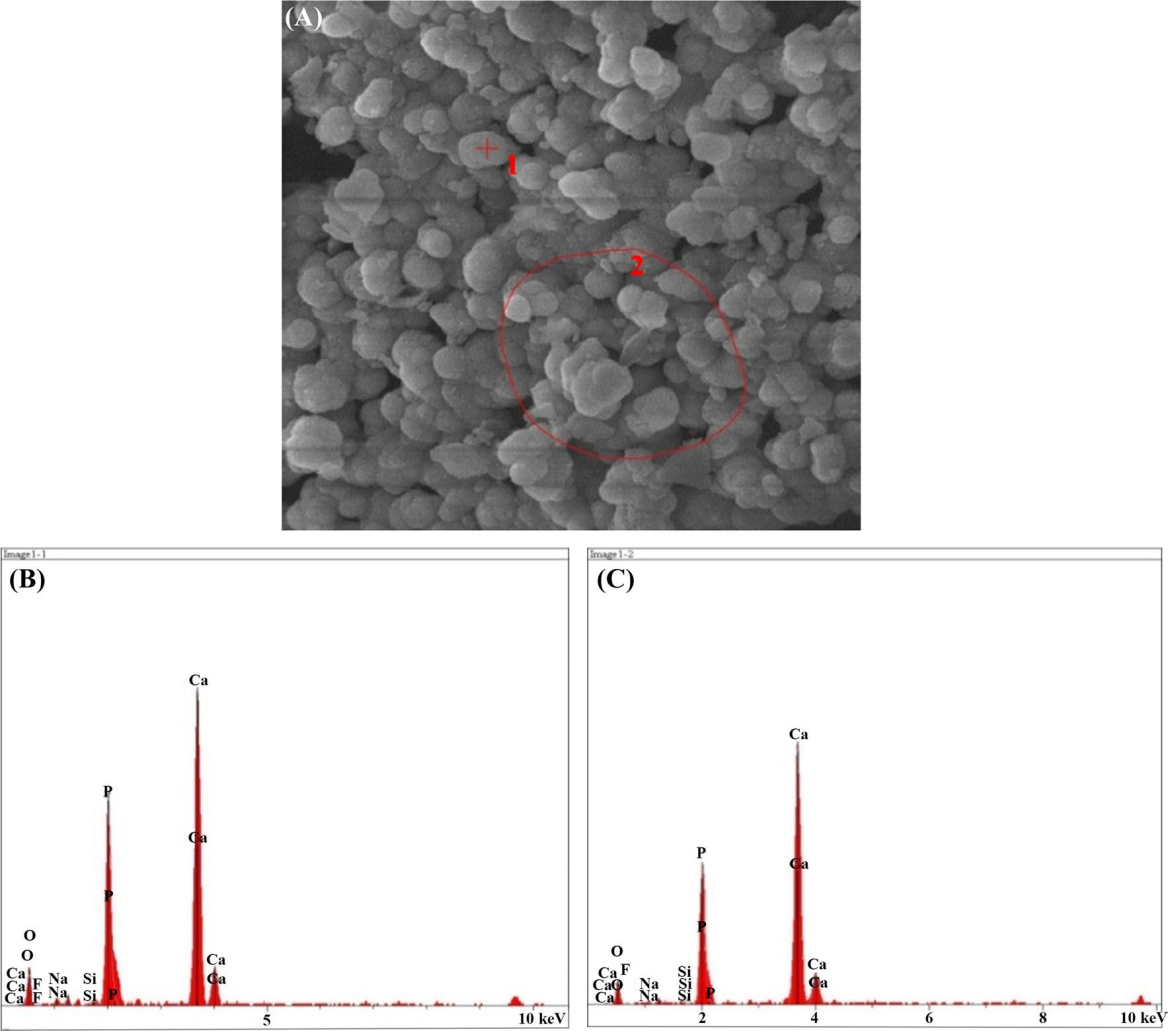
**a** SEM, **b**, **c** EDX images of the sample containing 15% fluorapatite

**Fig. 15:**
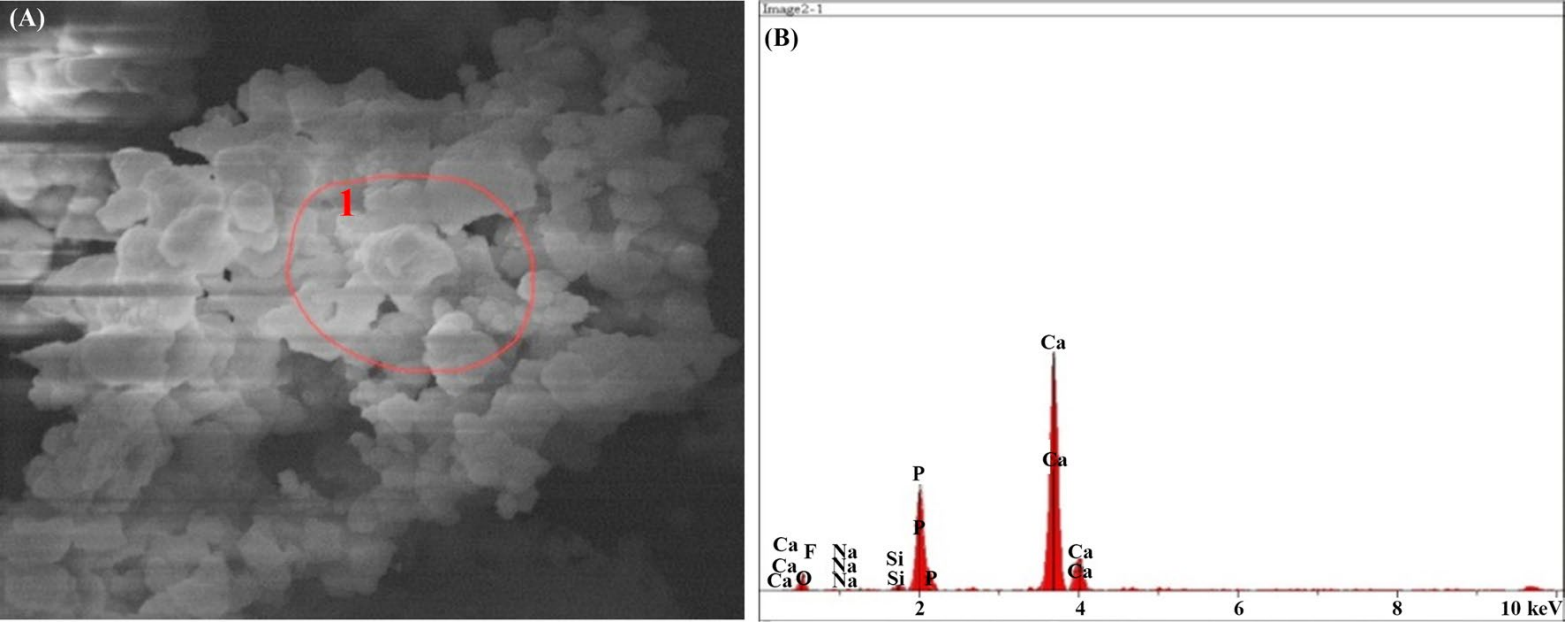
**a** SEM, **b** EDX images of the sample containing 20% fluorapatite

**Fig. 16:**
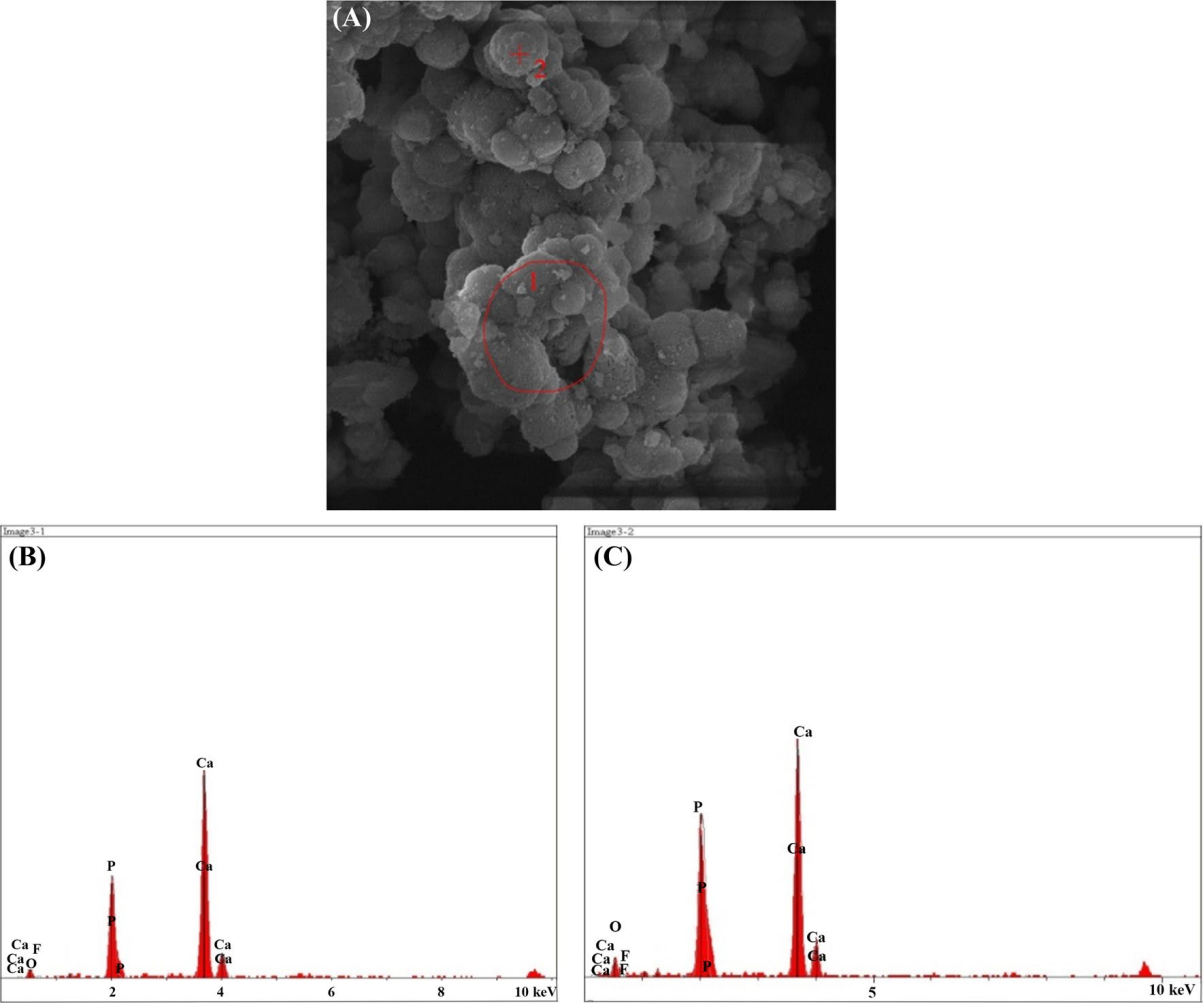
**a** SEM, **b**, **c** EDX images of the pure fluorapatite

The result of the elemental analysis of the particle beam energy spectrometry related to the synthesized powder samples is shown in the following Figs. 12, 13, 14, 15, 16. According to the X-ray diffraction pattern, there has been an increase in intensity of the peaks of the main elements in hydroxyapatite phase. This means that calcium and phosphorous exist in all samples and there is a reduction of Si in the samples containing the bioactive glass. These findings and the previous results would confirm the formation of apatite layers after the immersion of the sample in a body simulator solution, and as a result, the bioavailability of the produced samples was increased.

## Conclusion

Nano-bioactive glass of four apatite nanocomposites with 10%, 15%, and 20% fluorapatite was prepared by modified sol–gel method at 600 °C with particle size ranging between 20 and 50 nm. The crystallinity and the average diameter of the crystals were grown from 73 to 88% and 21 to 29 nm, respectively, when the fluorapatite percentages were increased from 10 to 20%. The bioactivity of the nanocomposites in a body-simulated fluid medium (SBF) was examined through the weight variation tests, pH fluctuations, and release of the silicon and the fluorine ions. The results indicated that the degradability increased by the addition of fluorapatite and the release of fluoride was promoted by increasing the apatite percentage. In addition, the silica release was increased in higher amounts of bioactive glass phase. In addition, all specimens exhibited an acceptable level of bioactivity and the apatite phase after 28 days of durability in the SBF solution, which was confirmed by EDS and SEM analyses.
